# Temporal changes in temperature-related mortality in relation to the establishment of the heat-health alert system in Victoria, Australia

**DOI:** 10.1007/s00484-024-02691-9

**Published:** 2024-05-06

**Authors:** Nicholas J. Osborne, Patrick Amoatey, Linda Selvey, Dung Phung

**Affiliations:** 1 School of Public Health, University of Queensland, Brisbane, QLD Australia; 2 Queensland Alliance for Environmental Health Sciences, University of Queensland, 266 Herston Rd, 4006 Herston, QLD Australia; 3 School of Population Health, University of New South Wales, Sydney, NSW Australia; 4 European Centre for Environment and Human Health (ECEHH), University of Exeter Medical School, Knowledge Spa, Royal Cornwall Hospital, Truro, Cornwall, UK

**Keywords:** Extreme temperature, Mortality, Heat-action plan, Victoria, Australia

## Abstract

Extreme heat alerts are the most common form of weather forecasting services used in Australia, yet very limited studies have documented their effectiveness in improving health outcomes. This study aimed to examine the temporal changes in temperature-related mortality in relation to the activation of the heat-health alert and response system (HARS) in the State of Victoria, Australia. We examined the relationship between temperatures and mortality using quasi-Poisson regression and the distributed lag non-linear model (dlnm) and compared the temperature-mortality association between the two periods: period 1– prior-HARS (1992–2009) and period 2– post-HARS (2010–2019). Since the HARS heavily weights heatwave effects, we also compared the main effects of heatwave events between the two periods. The heatwaves were defined for three levels, including 3 consecutive days at 97th, 98th, and 99th percentiles. We also controlled the potential confounding effect of seasonality by including a natural cubic B-spline of the day of the year with equally spaced knots and 8 degrees of freedom per year. The exposure-response curve reveals the temperature mortality was reduced in period 2 in comparison with period 1. The relative risk ratios (RRR) of Period 2 over Period 1 were all less than one and gradually decreased from 0.86 (95% CI, 0.72–1.03) to 0.64 (95% CI, 0.33–1.22), and the differences in attributable risk percent increased from 13.2 to 25.3%. The reduction in the risk of heatwave-related deaths decreased by 3.4% (RR_p1_ 1.068, 95% CI, 1.024–1.112 versus RR_p2_ 1.034, 95% CI, 0.986–1.082) and 10% (RR_p1_ 1.16, 95% CI, 1.10–1.22 versus RR_p2_ 1.06, 95% CI, 1.002–1.119) for all groups of people. The study indicated a decrease in heat-related mortality following the operation of HARS in Victoria under extreme heat and high-intensity heatwaves conditions. Further studies could investigate the extent of changes in mortality among populations of differing socio-economic groups during the operation of the heat-health alert system.

**Supplementary Information:**

The online version contains supplementary material available at 10.1007/s00484-024-02691-9.

## Introduction

The burden of disease and deaths attributable to extreme heat is increasing across different geographical scales because of climate change (Ebi et al. 2021; Zhao et al. 2021).

In the past three decades, populations across America, Australia, New Zealand, and Europe have been severely affected by the more frequent heatwaves expected to be attributed to anthropogenic climate change (Lemonsu et al. 2015; Chapman et al. 2017; Kotharkar and Ghosh 2022). In Australia, recent studies suggest that extreme heat is associated with an increase in the risk of ambulance demand, hospital admissions, and deaths, particularly among populations > 65 years and children under 5 years (Watson et al. 2020; Campbell et al. 2021; Franklin et al. 2023; Wondmagegn et al. 2021). These impacts have been observed in both the urban (Cheng et al. 2018; Guo et al. 2017; Hansen et al. 2008; Loughnan et al. 2010b) and rural areas (Loughnan et al. 2010a), strengthening the argument that Australian populations are vulnerable to extreme heat events regardless of the location. Regarding temporal changes in heat-related health effects, the study by Gasparini et al. (2015) assessed the changes mortality in Sydney and Melbourne city due to the low statistical power when comparing the effects between the two periods 1988–1998 and 1999–2009.

In response to rapidly rising number of extreme heat events, national and state governments have developed and operated heat-health warning systems (HHWS). These have been proven successful in reducing heat-related mortality in most high income nations, especially in some European countries (Martinez et al. 2022), the United States (Bobb Jennifer et al. 2014), and the United Kingdom (Lo et al. 2022). The effectiveness of the HHWS in improving health outcomes varies significantly by temporal changes in the temperature-response relationship, and health outcome among populations over time (Kotharkar and Ghosh 2022). For example, heat alerts may not necessarily decrease the risk of mortality, but could encourage individuals (especially those with fluid and electrolyte disorders) to seek medical care, suggesting increasing hospital admissions(Weinberger et al. 2021). Others have also found a high degree of heterogeneities about the effectiveness of HHWS in reducing mortalities among different regions(Díaz et al. 2019).

Assessment of the HHWS over time will provide information on their efficacy as a public health intervention tool during extreme weather events. Australia has seen an increase in frequency and severity of extreme heat events increasing over the last 100 years (Coates et al. 2014) and their associated health consequences such as fatalities and morbidities (Zhang et al. 2020; Coates et al. 2022). These record-breaking heat events have been observed in multiple cities in Australia making it an important public health concern (Beggs et al. 2021; Bi et al. 2011; Pezza et al. 2012). Although HHWS have been developed and operated in most jurisdictions in Australia in the last decade (Williams et al. 2019), no study has attempted to examine their effectiveness.

The Victorian Department of Health established a heat-health alert and response system (HARS) in 2009–2010 and reviewed its implementation in 2012–2013 (Williams et al. 2019). The HARS uses the forecast temperature to issue heat-health alerts for the specific district once the temperature exceeds the threshold which heat-related illness and mortality. This threshold is the 95th percentile 3 day moving average derived from 30 years of temperature data. This study aims to examine the temperature-related mortality over time including the HHWS intervention in Victoria, Australia over the 1992–2009 (prior-HARS) and 2010–2019 (post-HARS) time periods.

## Methods

### Research locations

**Fig. 1:**
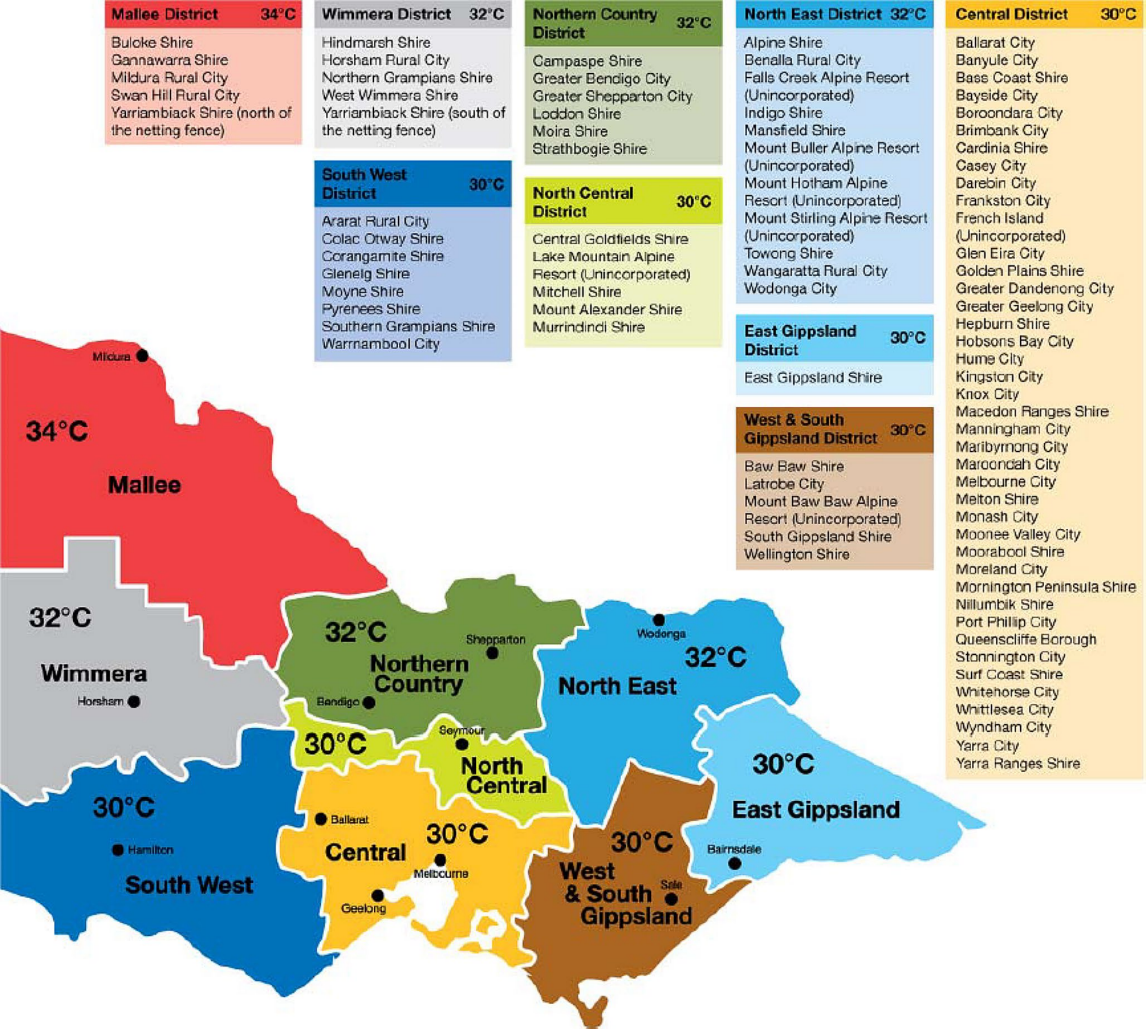
Weather forecast districts and corresponding heat health temperature thresholds. (Source: Extreme Heat Sub-plan for Victoria (2017))

This study was conducted in the state of Victoria in South-Eastern Australia. Victoria is the second-smallest State by land area of 227,44 km^2^, but it is the most densely populated state in Australia (28 per km^2^) with a total population of more than 6.5 million (Australian Bureau of Statistics 2021a). The state has 9 geographical districts including 96 cities and counties (Fig. 1). The climate of this state ranges from semi-arid temperature with hot summers in the northwest, to temperate and cool along the coastal areas (Beck et al. 2018). The temperatures in summer range between 14 and 25.3 °C (December-February). According to Loughnan et al. (2010a), extreme heat is considered one of the most significant health hazards facing Victoria, and it causes exacerbation of pre-existing conditions in the population and may result in increased propensity of heat-related mortality among vulnerable populations especially the elderly (> 65 years). For example, 374 and 167 excess deaths were recorded during the bushfire-induced heatwaves in 2009 and 2014 in Victoria (Victoria Government 2020).

### Data

Daily count of all-cause death data for Victoria residents during January 1996-December 2015 (inclusive) were obtained from the Australian Bureau of Statistics (ABS). This mortality data covers nearly 78.5% of Victorian population living in central part of the State. The data includes the number of deaths for all causes for the State of Victoria by date of death, sex, and age groups (0–15, 16–64, 65–74, 75–84, and 85+).

Data on temperatures were obtained from the Bureau of Meteorology for the Melbourne Airport weather station (144.844788,-37.663712) which locates in the city of Melbourne in the southern part of the central forecast district area of Victoria, where 5.1 million of 6.5 million Victorian residents reside. The data includes daily maximum and minimum temperatures from 1st Jan 1992 to 15th December 2019. To evaluate the temporal change in heat-related mortality in relation to the HARS operational date, we used the average temperature which was calculated from the maximum temperature of one day and the minimum temperature for the following day (Extreme Heat Sub-plan for Victoria 2017). The Victorian Health Department is using this calculation method using the forecast temperatures for HARS (Extreme Heat Sub-plan for Victoria 2017).

The heat-health plan for Victoria (HHP-V) (Victoria Government 2020), which has been developed since 2009–2010, guides how the State Department of Health and Human Services works together with local government and health and human services sectors to promote public health and wellbeing before and during periods of extreme heat (3 days of 95th percentile temperatures). The HHP-V has three main objectives: (i) protect the health of Victorians from heat-related harm; (ii) communicate the actions of the department to prepare for and respond to extreme heat; (iii) outline preparedness and response activities for local government and the health and human services sectors to reduce the impact of extreme heat on Victorians (Victoria Government 2020). The key element of the HHP-V is the HARS which has been developed to notify the stakeholders (local councils, departmental program areas, hospitals, and health and community service providers) that the average temperature is predicted to reach and exceed heat-health thresholds. The thresholds are identified by a range of evidence and information and vary across Victoria to recognize the higher temperatures experienced in its northern parts. A heat health temperature threshold has been established for each of the nine weather forecast districts (Fig. 1). The average temperature for any given day is calculated as the mean of the forecast daily maximum temperature and the forecast overnight minimum temperature (which is the daily minimum for the following day). When the forecast average temperatures are predicted to reach or exceed the heat health temperature threshold of 30–34℃ depending on specific weather forecast district, the State government department would issue a heat health alert for that district. Among the most common actions taken by the department during extreme heat days includes media notification/release, messaging (via digital platforms and via radio, television), monitoring of health system demands, involvement of local community organization to increase heat-health information dissemination, ensures the availability of adequate cool spaces/water, and monitoring of weather systems (Victoria Government 2020).

### Statistical analysis

We examined the relationship between the temperature and mortality counts using quasi-Poisson regression and the distributed lag non-linear model (DLNM ) framework (Gasparrini 2014). This method is warranted as it captures the non-linear and delayed effects (lagged association) which are the typical characteristics of heat-health response, using basis functions (Martínez-Solanas and Basagaña 2019). Since the HARS was established and operated in 2009–2010, we conducted data analysis separately for two periods, period 1 (1992–2009) and period 2 (2010–2019).

The exposure-response association was regressed using a quadratic B-spline with 3 internal knots placed at the 10th, 75th, and 90th percentiles of temperature distribution (Ngandu et al. 2015). The potential confounding effect of seasonality was controlled by including a natural cubic B-spline of the day of the year with equally spaced knots and 8 degrees of freedom per year. An interaction between this spline function and the variable of the year was specified to relax the assumption of a constant seasonal trend. We controlled the effects of long-term trends and the week’s days by incorporating a linear term for a year and the variable for a day of the week into the model. The lag-response association was modelled using a natural cubic B-spline with an intercept and three internal knots placed at equally spaced values in the log scale. The lag period was extended up to 21 days including possible long delays in the effects of the temperature (Martínez-Solanas and Basagaña 2019; Davis et al. 2023). The overall cumulative effects of temperature for all lags were then plotted to visually examine the difference in the temperature-mortality association between the two periods.

To compare the temperature-mortality association between the two periods, we estimated the relative risk (RR) and an attributable fraction (AF) for the temperatures above 30^o^C, which was the lowest temperature threshold used for the HARS, relative to the reference temperature at 22.5^o^C that was identified from the previous study (Gasparrini et al. 2015). The relative risk ratio (RRR) and differences in AF between the two periods were then estimated. Since the HHP-V heavily weights heatwave effects, we also compared the main effects of heatwave events between two periods. The heatwaves were defined for three levels, including 3 consecutive days at 97th, 98th, and 99th percentiles which are in line with the heatwave definitions by the HHP-V (Victoria Government 2020; Tong et al. 2015). The main effects of heatwaves were calculated using the models using the same cross-basis function of the average temperature described above. The details on how to quantify the main effects of heatwaves were described elsewhere (Gasparrini and Armstrong 2011). The comparison of the effects between the two periods was analyzed by sex and age groups (16–74, 75+). The analyses on two heatwave periods ( period 1:1992–2009, and period 2:2010–2019) were restricted to the summer only (December to February), a major season where heat-related deaths normally occur in Australia (Coates et al. 2014).

We conducted several sensitivity analyses to examine the robustness of our findings. First, we determine how significant of a break point the implementation of the current HARS periods. We applied the same study design, and arbitrarily defined HARS at different periods of implementation: (i) 1992–2005 as period 1– prior-HARS, and (ii) 2006–2019 as period 2– post-HARS implementation at lag 21 days. Second, we examined whether changes in temperature-mortality relationships were not underestimated between period 1 (1992–2009), and period 2 (2010–2019) by expanding the analyses at three different shorter lag periods (i.e. lag 4 days, lag 7 days, lag 14 days), since adverse health effects of heatwave normally last for few days. In both analyses, heatwaves were defined for three levels, including 3 consecutive days at the 97th, 98th, and 99th percentiles (Benmarhnia et al. 2016a). All analyses were performed with R (version 4.1.2) and Stata (version 17).

## Results

### Descriptive statistics

**Fig. 2:**
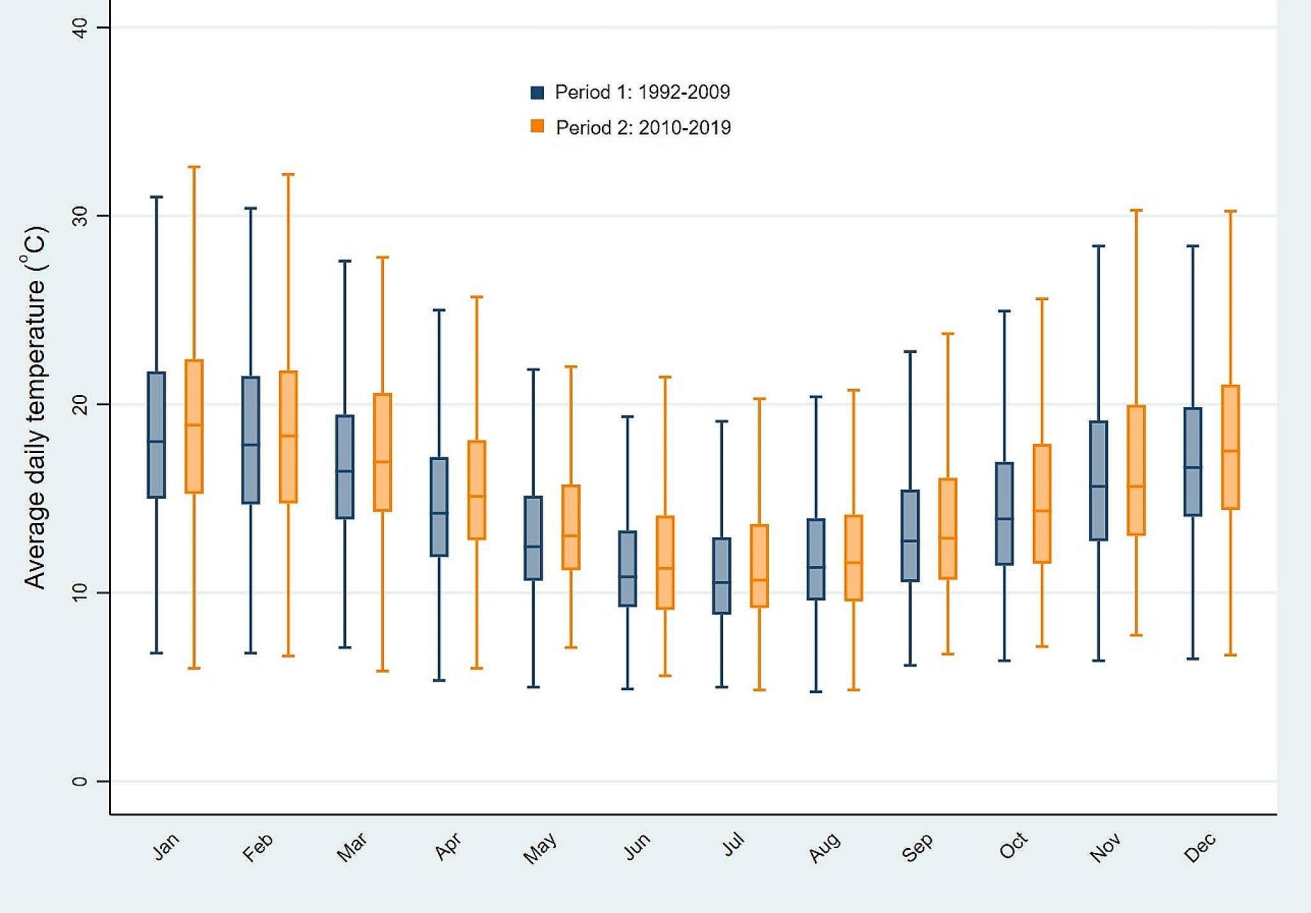
Distribution of the average temperature by period

The descriptive statistics of the study population and temperatures by period are shown in Table 1. The total number of deaths that occurred between 1992 and 2019 was 978,690 with a mean of 96 (SD, 15) deaths per day. The average number of daily deaths (ANDD) was higher in period 2 (105 ± 14 versus 91 ± 13), reflecting an increase in the total population (an increase of 2,138,661 inhabitants during the study period) (Australian Bureau of statistics 2019). While the ANDD was stable for the age group of 16–74, it was higher in period 2 for the older people at 75-year-old and above (72 ± 11 versus 56 ± 11). In terms of sex, the daily counts of mortality were equally higher in period two for both males and females. The temporal decrease in the mortality rate over time in period 1 (-0.11/1,000,000 persons/ year) was approximately similar to that observed in period2 (-0.1/1,000,000 persons/year). Both the means of daily average maximum and minimum temperatures were higher in period 2 (7.9 °C versus 8.3 °C and 19.9 °C versus 20.6 °C) (Table 2). This is also seen in the temporal pattern of the average daily temperature between two periods shown in Fig. 2.

**Table 1:** Descriptive Statistics

Variable	Period 1 (1992–2009)			Period 2 (2010–2019)		
(a) Mortality (counts)	Min	Mean (SD)	Max	Min	Mean (SD)	Max
All	5	91 (13)	268	46	105 (14)	184
Male	1	47 (8)	146	19	53 (9)	93
Female	0	44 (8)	122	21	52 (9)	101
Age 16–74	0	19 (5)	154	2	18 (5)	36
Age 75+	3	56 (11)	122	25	72 (11)	132
(b) Temperature						
Daily maximum temperature (℃)	7.9	19.9 (6.4)	46.8	8.3	20.6 (6.7)	46
Daily minimum temperature (℃)	-1.2	9.6 (4.3)	30.5	-2	9.9 (4.5)	28.8

Min: minimum, Max: maximum, SD: standard deviation

### Change in the temporal pattern of the temperature-mortality association in the two periods

**Fig. 3:**
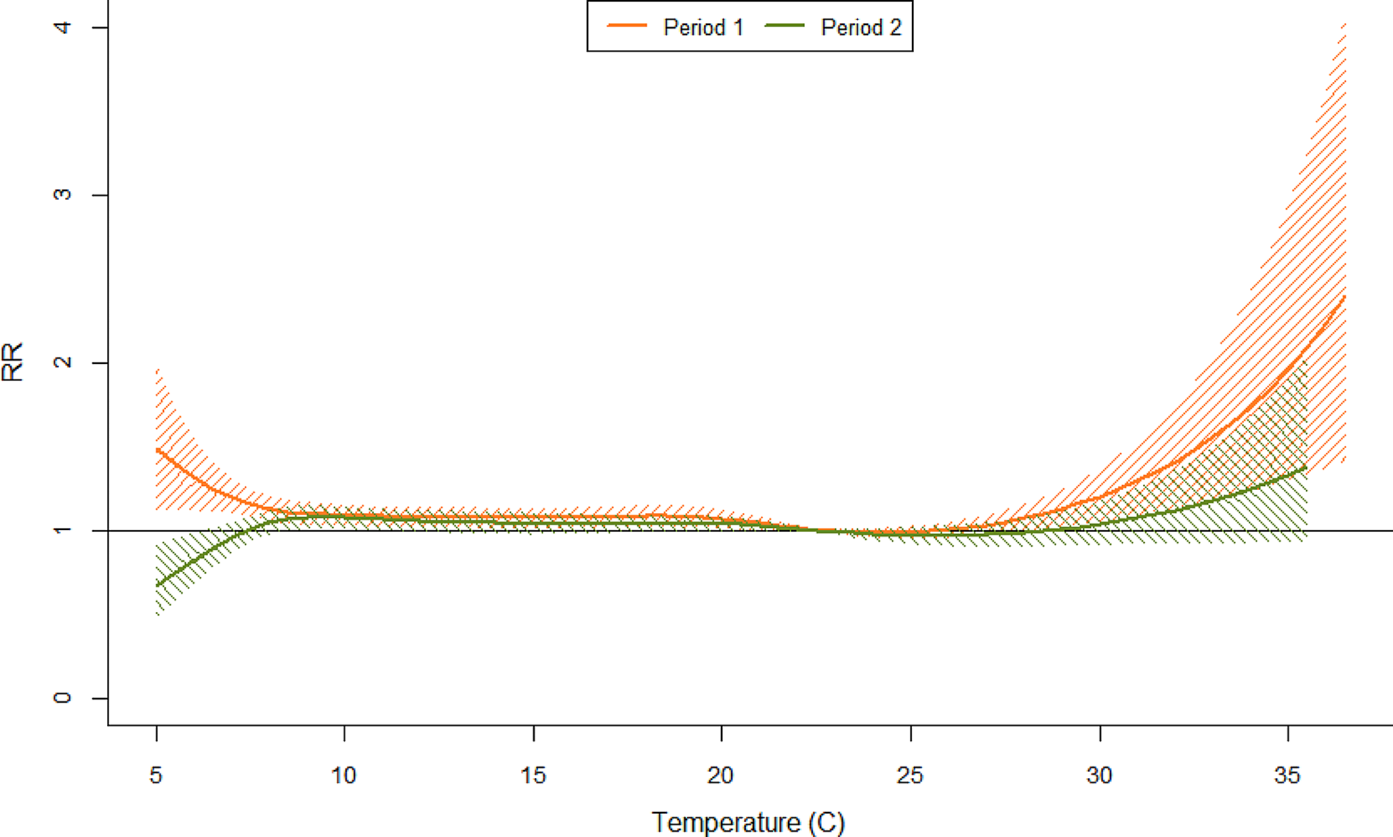
Overall cumulative temperature-mortality relationship for the two study periods (1992–2009 and 2010–2019). Shaded areas correspond to a 95% confidence interval

The pattern of the relationship between average temperature and the risk of death in the two periods is shown in the overall cumulative exposure-response curve in Fig. 3. For both periods, the curves had a J-shape, reflecting a rapid increase in risk of mortality as average daily temperaturees increased. The risk of deaths associated with cold temperatures was reduced substantially, especially the effect of extreme cold at a temperature less than the 5th percentile (8.2^o^C) disappeared in period 2 while this effect was found significant in period 1. For the effect of higher temperatures, the slope of the risk of mortality curve was reduced in period 2 in comparison with period 1 although the confidence intervals for the higher temperatures in the two periods overlapped. The threshold of the elevated temperature-mortality was observed at 29^o^C which was significantly higher than the threshold observed in period 1 (27^o^C). It is noteworthy that the thresholds of both periods were lower than the minimum threshold (30^o^C) used for the activation of HARS in Victoria.

The comparison of relative risk and attributable fraction were calculated for the average temperatures from 30^o^C up to the maximum relative to the reference temperature at 22.5^o^C (Table 1). The risk of deaths statistically increased monotonically by the increase in the temperature for period 1; whereas this linear effect was not statistically significant observed for period 2 (all 95% CI of RRs included the unity) (Table 2). The relative risk ratios (RRR) were all less than one and gradually decreased from 0.86 (95% CI, 0.72–1.03) to 0.64 (95% CI, 0.33–1.22), however the RRRs were not statistically significant, reflecting the overlapped 95% CI presented in Fig. 3. The differences in attributable risk percent increased from 13.2 to 25.3% as shown in Table 2.

**Table 2:** Relative risk, relative risk ratio, differences in attributable risks (AR), and attributable risk percentage (AR%) of temperature-mortality between two periods

Temperature (°C)	Period 1 (1992–2009) RR_p1_ (95% CI)	Period 2 (2010–2019) RR_p2_ (95% CI)	Relative Risk Ratio (RR_p2_/RR_p1_)(95% CI)	Difference in AR% (AF_p2_– AF_p1_)
30	1.20 (1.06–1.36)	1.04 (0.92–1.17)	0.86 (0.72–1.03)	-13.2
31	1.29 (1.09–1.53)	1.07 (0.92–1.25)	0.83 (0.66–1.04)	-15.8
32	1.41 (1.14–1.75)	1.12 0.93–1.25)	0.79 (0.59–1.06)	-18.3
33	1.55 (1.18–2.04)	1.18 (0.93–1.49)	0.76 (0.53–1.09)	-20.6
34	1.73 (1.24–2.43)	1.25 (0.93–1.67)	0.72 (0.46–1.12)	-22.5
35	1.96 (1.30–2.94)	1.33 (0.93–1.89)	0.68 (0.39–1.17)	-24.1
36	2.24 (1.38–3.63)	1.43 (0.93–2.19)	0.64 (0.33–1.22)	-25.3

The reference temperature is 22.5°C

**Fig. 4:**
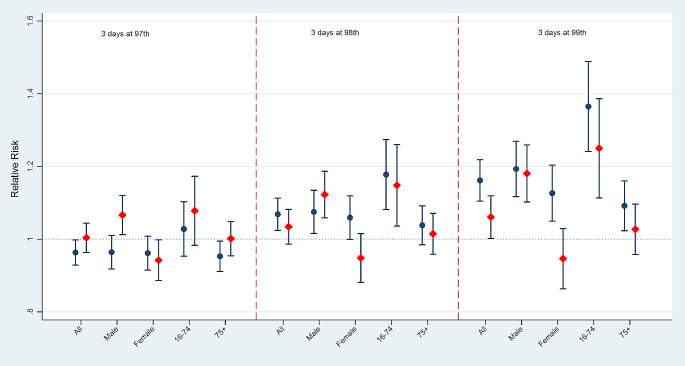
The main effect of heatwaves on the risk of death for two periods. The blue circle and the red diamond present the relative risk for period 1 and period 2, respectively. The vertical line presents 95% CI.

Figure 4 presents the main effects of heatwaves for two periods. The main effect of the heatwave was calculated as the risk ratio between the median temperatures during the heatwave duration and the 75th percentile of the temperature outside the heatwave durations. The heatwave defined at the 97th percentile did not cause significant health effects for almost all the groups (except males). Heatwaves defined at the 98th and 99th percentiles, reductions in the risk of heatwave-related deaths were observed for all groups except males from period 1 to period 2. The risks of death for the general population decreased by 3.4% (RR_p1_ 1.068, 95% CI, 1.024–1.112 versus RR_p2_ 1.034, 95% CI, 0.986–1.082) and 10% (RR_p1_ 1.16, 95% CI, 1.10–1.22 versus RR_p2_ 1.06, 95% CI, 1.002–1.119) for all groups of people. It is noteworthy that the risks declined from statistically significance (at the 98th percentile: RR_p1_ 1.059, 95% CI, 1.0-1.12; at the 99th percentile: RR_p1_ 1.13, 95% CI, 1.05–1.20) in the period 1 to non-significance in period 2 (at the 98th percentile: RR_p2_ 0.95, 95% CI, 0.88–1.02; at the 99th percentile: RR_p2_ 0.95, 95% CI, 0.86–1.23) among female groups, and this was also observed among elderly (75+) at the 99th threshold heatwave (RR_p1_ 1.09, 95% CI, 1.02–1.16 versus RR_p2_ 1.03, 95% CI, 0.96–1.10). The reductions were also observed in the people aged from 16- to 74-year-olds (at the 98th percentile: RR_p1_ 1.18, 95% CI, 1.08–1.27 versus RR_p2_ 1.15, 95% CI, 1.04–1.26; at the 99th percentile: RR_p1_ 1.37, 95% CI, 1.24–1.49 versus RR_p2_ 1.25, 95% CI, 1.11–1.39).

### Sensitivity analysis

The adjusted cumulative temperature-mortality relationship and the differences in attributable risk percent for the selected arbitrary two study periods in the sensitivity analysis are shown in Supp. Figure 1, and Supp. Table 1, respectively. We compared these two arbitrary periods (1992–2005 and 2006–2019) which were unrelated to the actual HARS implementation periods. We did not find evidence of a decreasing risk of mortality following the implementation of the latter period. As shown in Supp. Figure 1, the slope of the risk of mortality curve rather increased in period 2 in comparison with period 1 during high temperatures, when compared to actual implementation periods. Similarly, the relative risk ratios (RRR) of heat-related deaths were all greater than 1 and sharply increased from 1.19 (95% CI, 0.76–1.88) to 2.07 (95% CI, (0.84–5.12), the RRRs were found to be statistically significant at 95% CI (Table 3). The Supp. Figure 2 and Supp. Tables 2–4, shows the sensitivity analysis of the effect of different lag structures (lag 4 days, lag 7 days, and lag 14 days) on cumulative temperature-mortality relationships in period 1 (1992–2009) and period 2 (2010–2019). We observed that the reduction in heat-associated death increases with increasing lag days following post-HARS activation (2010–2019).

## Discussion

This study examined the association between extreme temperatures and mortality in Victoria, Australia in two periods (1992–2009 and 2010–2019). The heat-health alert and response system (HARS) were established and operated in 2009–2010. The results of the study provide the implication for the efficacy of the HARS since we compared the pattern and absolute effects of temperatures between two periods. These results are robust to sensitivity analysis as the risk of mortality curve did not increase in period 2 (arbitrary period 2006–2019) in comparison with period 1 (arbitrary period 1992–2005) during high temperatures, compared to actual implementation periods. Overall, we observed the reductions in risk of deaths associated with the extreme temperatures in period 2 compared with period (1) The reductions were higher corresponding to the higher threshold of the temperatures. In addition to the decrease in the heat-related risk of death, the effect of extreme cold in the summer period disappeared in period (2) However, despite the obvious reduction pattern in heat-related mortality, the risk ratios between the two periods were not statistically significant. We interpreted the risk ratios without accounting for statistical significance because we considered p-value alone as not an objective measure and did not provide enough information about hypothesis testing (Baghi et al. 2007; Hayat 2010). For example, similar studies have also concluded the effectiveness of heat action plans in reducing heat-related mortality without considering statistical significance (Díaz et al. 2019; Ebi et al. 2004).

The findings of this current study increase the evidence on the benefits of the heat-health action plan, which includes the HARS as a key component, in reducing heat-related mortalities across countries worldwide. A significant reduction in heat-related mortality was observed in the cities in Italy (Schifano et al. 2012), Montreal in Canada (Benmarhnia et al. 2016b), and Paris in France (De’Donato et al. 2015). Although there is no study comprehensively evaluating the efficacy of heat-health action plans and HARS in Australia, the previous studies looked at the public perception and responses toward heat health warnings in regional areas. The study by William et al. (2018) indicated that the heat-health warnings had been well received and understood, however the behavior change was varied by regional populations in Australia. Thus, this study encourages reinforcing existing protective behavior rather than promoting change (Williams et al. 2019). The findings also found that women had higher perceptions of a heat-related health risks than men. This is an interesting point and well reflected by the significant reduction of heat-related mortality among women observed in our study in comparison with other groups, although it may reflect the propensity of cardiovascular diseases in males in this population. It may also reflect the sexual dimorphism in ratio of occupations involving outdoor work (Fatima et al. 2021). Another study by the Williams et al. (2021) estimated that the potential cost savings from reduced morbidity offset the estimated heat-health warnings implementation costs by at least two-fold (Williams et al. 2021). Both studies encouraged the effectiveness of the systems should be further evaluated.

For example, evaluating HARS according to pre- and post-implementation in the case of this study may capture confounding factors that are likely to change over time such as increasing cognizance of heat-health education, air conditioning prevalence rate, and modern housing systems. In addition, behavioral change toward adaptations (e.g., taking annual leave during the summer season), and an inherent decrease in heat sensitivity because of acclimatization may not be linked with HARS implementation(Weinberger et al. 2021; Boeckmann and Rohn 2014). On the other hand, an increase in migration may increase the risk of heat-related outcomes as new migrants may lack acclimatization or have limited knowledge of accessing emergency healthcare services during extreme heat events(Hansen et al. 2014). Also, improvement in socioeconomic conditions may be predictors that can decrease the risk of heat-related deaths(Ng et al. 2016). Evidence shows that Victoria has shown an improvement in the unemployment rate (5%), and increased number of home ownership (36.1%) in 2021 compared to 2006 where they were 5.4%, and 34%, respectively (Australian Bureau of Statistics 2006, 2021b). Having recognized these potential confounding factors, we can then understand the true effectiveness of HARS in Victoria by comparing days of activation of the heat alerts versus days without heat alerts (Weinberger et al. 2021).

In terms of other potential mechanisms of the heat-health action plan for reducing heat-related health risks, the evidence from the previous studies suggests that the plans could reduce risks through surveillance and monitoring of effects (Elliot et al. 2014; Perry et al. 2011; Josseran et al. 2009), mobilizing of community resources (Hasan et al. 2021). The influence of the plans may change over time and vary by location, thus they should be developed to meet the local needs and based on the latest evidence of health risks as well as the effectively existing preventive measures which could be readily implemented by the local public health officials and community (Jay et al. 2021). Our study provides updated evidence on the temperature threshold as well as the temporal changes in mortality in relation to the operation of the heat-health action plan and alert system at the State level of the Victoria suggests positive benefits from use of the action plan. We found important evidence on the efficacy of the heat-health alert system in reducing heat-related mortality among women and vulnerable groups such older people. Our sensitivity analysis confirms that heat-associated death reduces few weeks after the activation of the heat-health action plan and alert system. This suggest that the implementation period effectively reduces risk of death and could be helpful in future risk communication.

The lower threshold temperature observed in this study warrants further research to determine the updated optimum temperature thresholds used for the specific locations in the alert system. Similar to previous studies, this current study encounters the limitation in evaluating whether the reduction in heat-related mortality is due to the heat-health alert system itself or a growing understanding of the risk of high heat exposure in the community and the implementation of mitigation factors in the community. These may include factors such as improved housing conditions (air conditioning, design), and increasing greenspaces in better designed newer suburban areas.

This study has some limitations. First, our study used the average temperature registered from a single monitoring station in the central area, so the exposure of temperature might be misclassified in rural areas that are far from the central area. However, the proportion of the rural populations is small (approximately 12%), thus the potential bias in overall estimates is expected to be small. Second, we were unable to obtain the mortality data by districts and regions but aggregated data for the whole of Victoria, so the evaluation was not conducted against the temperature thresholds which vary by the region. There are significant differences in average daily temperature with increases seen in areas more continental areas, away from the coast. Alternatively, we examined the temporal change of mortality in relation to the temperature before and after the implementation of the heat-health alert system at the state level. A future study should examine the effectiveness of the heat-health alert system at a higher resolution level since the heat-health alerts are issued for specific local governments once the temperature thresholds of the relevant districts have exceeded the threshold. Third, the design of our study does not allow us to assess the causality because we did not have a control group and data on heat-health adaptation in the household- and individual-level (e.g., increase in the prevalence of mitigation factors such as air conditioning over time). A more sophisticated study design, such as difference-in-difference, can address the effect of underlying time-dependent trends in the health outcomes unrelated to the heat-health intervention. The populations overall understanding of the risk of heat as part of a changing environment has also changed during this time and the contribution of this (as opposed to the HHWS alone) is unclear.

## Conclusion

The study indicated a decrease in mortality attributed to high ambient temperatures and heatwaves after the operation of the heat-health alert and response system (HARS) in Victoria. The decrease was observed more with high intensity of heatwaves. The temperature thresholds are likely to change over time, so further studies should be conducted to investigate this change to adapt the heat-health alert system and enhance the public health actions in reducing the heat-related health risk in a timely fashion.

### Electronic supplementary material

Below is the link to the electronic supplementary material.

Supplementary Material 1
