# Supplementary material for: Temporal changes in temperature-related mortality in relation to the establishment of the heat-health alert system in Victoria, Australia
Source: Int J Biometeorol. 2024 May 6;68(8):1637–47. doi: 10.1007/s00484-024-02691-9 (PMC11282152; doi:10.1007/s00484-024-02691-9)
Supplement: Supplementary file 1 — Supplementary Material 1 [file 484_2024_2691_MOESM1_ESM.docx]

**Temporal changes in temperature-related mortality in relation to the establishment of the heat-health alert system in Victoria, Australia**

Nicholas J. Osborne^1,2,4^, Patrick Amoatey ^1^, Linda Selvey^1^, Dung Phung ^1,2*^

1. School of Public Health, University of Queensland, Brisbane, Queensland, Australia.
2. Queensland Alliance for Environmental Health Sciences, University of Queensland, Australia.
3. School of Population Health, University of New South Wales, Sydney, NSW, Australia.
4. European Centre for Environment and Human Health (ECEHH), University of Exeter Medical School, Knowledge Spa, Royal Cornwall Hospital, Truro, Cornwall, UK

* Corresponding author

Dung Phung

Phone: +61(07)33464684

Email: d.phung@uq.edu.au

Address: 266 Herston Rd, Herston QLD 4006, Queensland, Australia

Supplemental File

**Sensitivity Analysis**

The adjusted cumulative temperature-mortality relationship and the differences in attributable risk percent for the selected arbitrary two study periods in the sensitivity analysis are shown in Supp. Figure 1, and Supp Table 1, respectively. We compared these two arbitrary periods (1992-2005 and 2006-2019) which were unrelated to the actual HARS implementation periods. We did not find evidence of a decreasing risk of mortality following the implementation of the latter period. As shown in Supp Figure 1, the slope of the risk of mortality curve rather increased in period 2 in comparison with period 1 during high temperatures, when compared to actual implementation periods. Similarly, the relative risk ratios (RRR) of heat-related deaths were all greater than 1 and sharply increased from 1.19 (95% CI, 0.76-1.88) to 2.07 (95% CI, (0.84-5.12), the RRRs were found to be statistically significant at 95% CI (Table 3).

The sensitivity analysis was robust to the main findings when the lag of 21 days was reduced to shorter lag days (lag 4. Lag 7 and lag 14). As shown in Supp Figure 2, the slope of the risk of mortality curve increased in period 1 (1992-2009) in comparison with period 2 (2010-2019) during high temperatures for all the different lag structures.The relative risk ratios (RRR) of Period 2 over Period 1 were all less than one under lag 7 days and lag 14 days, and the differences in attributable risk percent increased from 6.2% to 9.1%, and from 16.4% to 24.4%, respectively (Supp. Table 3-4).


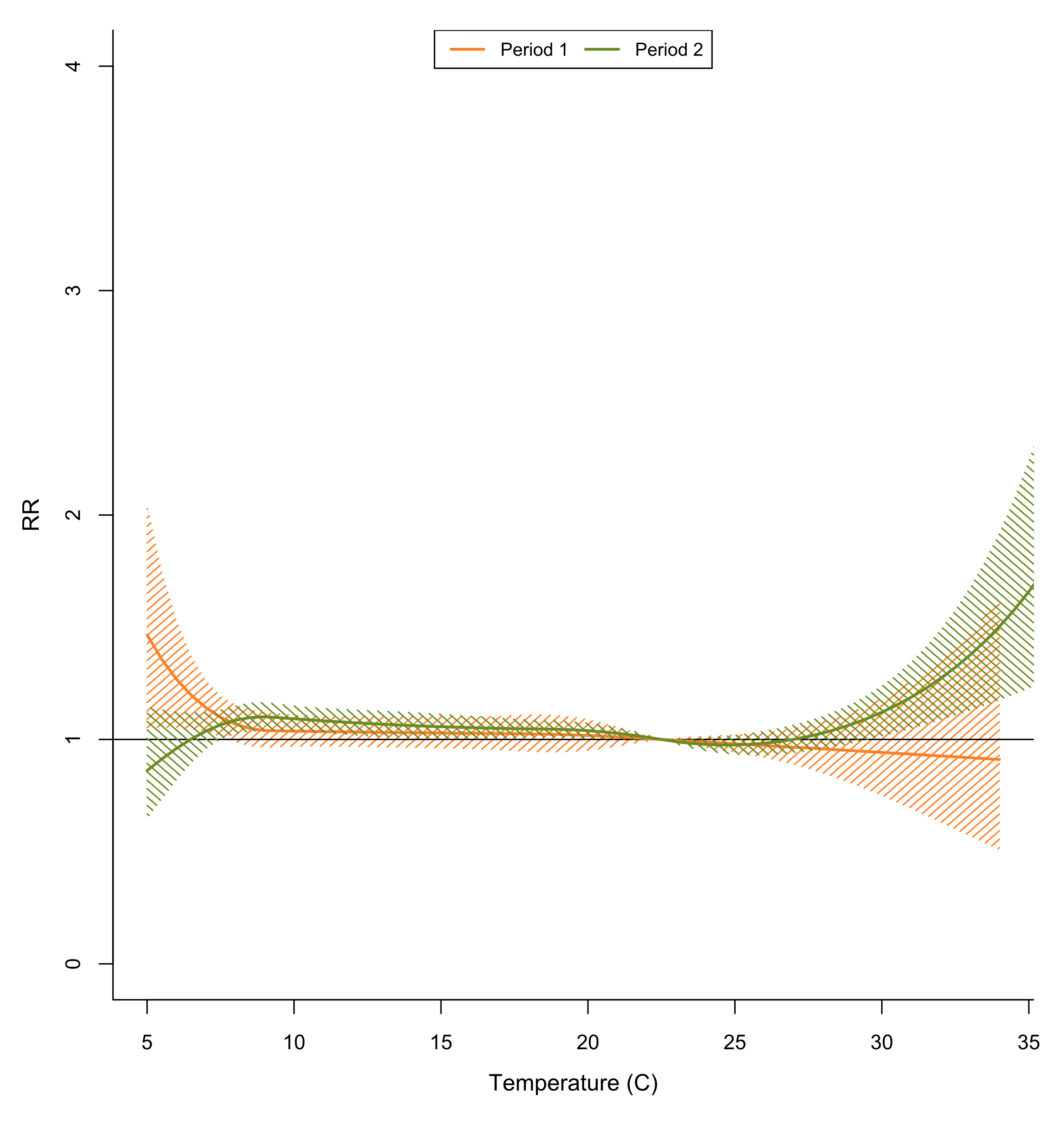


Supp. Figure 1. Sensitivity analysis of overall cumulative temperature-mortality relationship for the two study periods (1992-2005 and 2006-2019). Shaded areas correspond to a 95% confidence interval.

Supp. Table 1. Sensitivity analysis for relative risk, relative risk ratio, differences in attributable risks (AR), and attributable risk percentage (AR%) of temperature-mortality between two periods.

| Temperature  (°C) | Period 1 (1992-2005)  RR_p1_ (95% CI) | Period 2 (2006-2019)  RR_p2_ (95% CI) | Relative Risk Ratio  (RR_p2_/RR_p1_)(95% CI) | Difference in AR%  (AF_p2_ – AF_p1_) |
| --- | --- | --- | --- | --- |
| 30 | 0.94 (0.75 – 1.82) | 1.12 (1.02 – 1.24) | 1.19 (0.76-1.88) | +17.1 |
| 31 | 0.93 (0.69 – 1.26) | 1.89 (1.05 – 1.34) | 2.03 (1.47-2.81) | +54.6 |
| 32 | 0.93 (0.63 – 1.36) | 1.27 (1.09 – 1.49) | 1.37 (0.90-2.07) | +28.8 |
| 33 | 0.92 (0.57 – 1.48) | 1.38 (1.13 – 1.67) | 1.50 (0.90-2.51) | +36.2 |
| 34 | 0.91 (0.51 – 1.63) | 1.50 (1.78 – 1.92) | 1.65 (0.92-2.95) | +43.2 |
| 35 | 0.90 (0.45 – 1.81) | 1.66 (1.23 – 2.24) | 1.84 (0.86-3.93) | +50.9 |
| 36 | 0.89 (0.39 – 2.04) | 1.85 (1.29 – 2.65) | 2.07 (0.84-5.12) | +58.3 |

The reference temperature is 22.5^o^C.


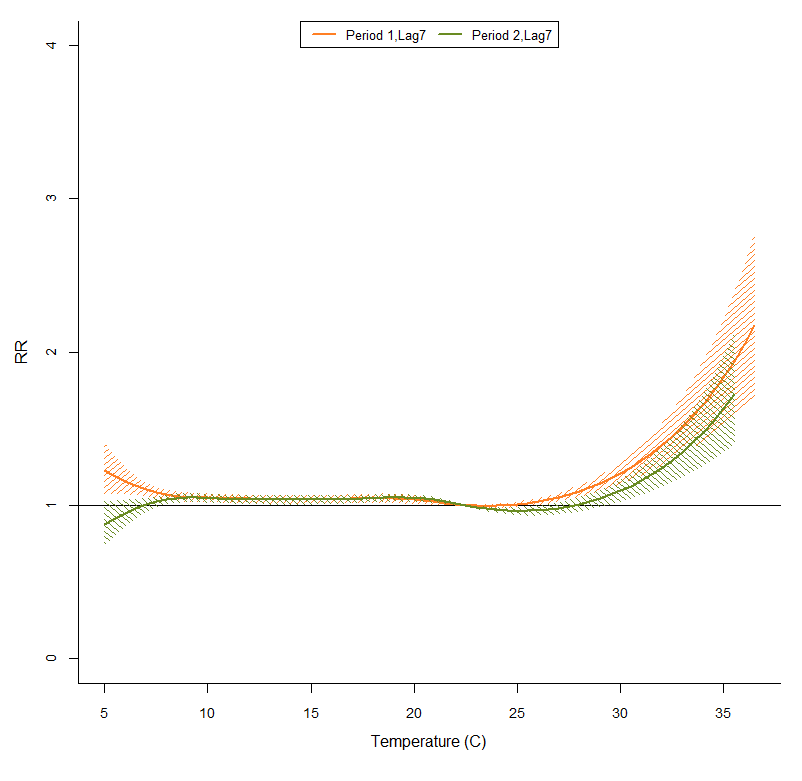

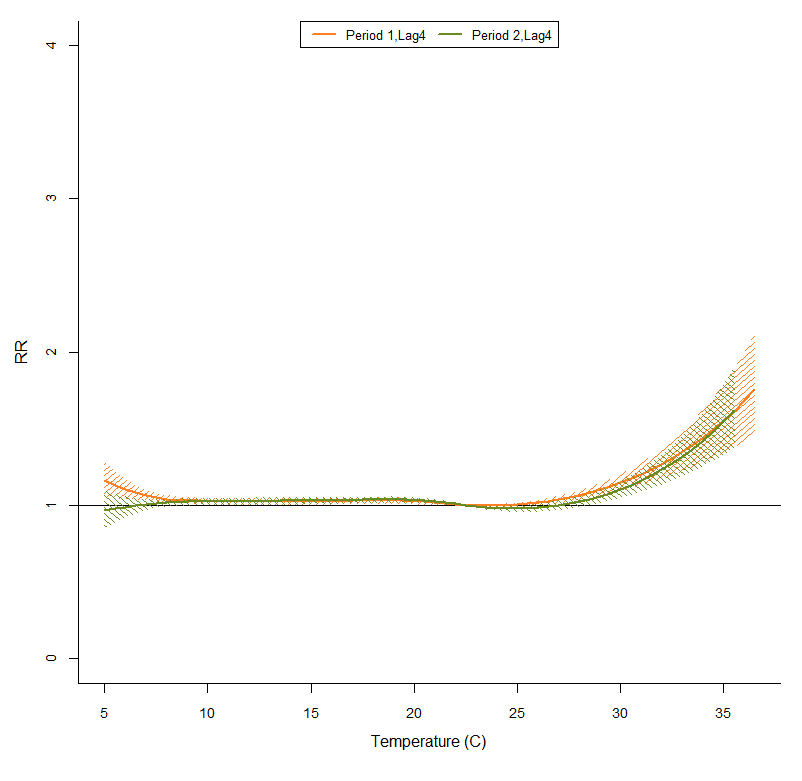

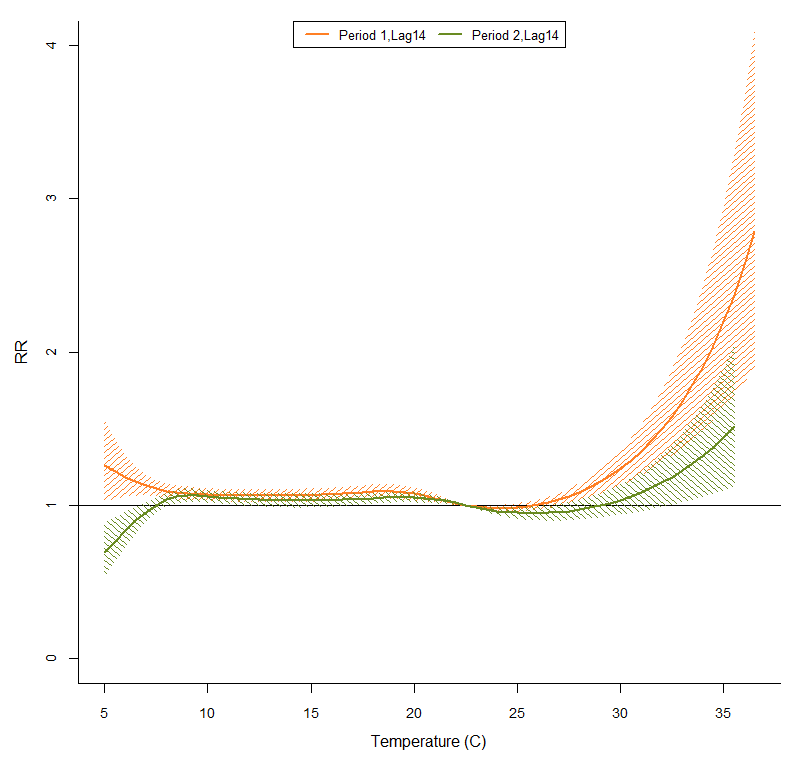


(a)

(b)

(c)

Supp. Figure 2. Sensitivity analysis of overall cumulative temperature-mortality relationship for the two study periods (1992-2009 and 2010-2019) at (a) lag 4 days, (b) lag 7 days, and (c) lag 14 days. Shaded areas correspond to a 95% confidence interval.

Supp. Table 2. Lag 4 days sensitivity analysis for relative risk, relative risk ratio, differences in attributable risks (AR), and attributable risk percentage (AR%) of temperature-mortality between two periods

| Temperature  (°C) | Period 1  (1992-2009)  RR_p1_ (95% CI) | Period 2  (2010-2019)  RR_p2_ (95% CI) | Relative Risk Ratio  (RR_p2_/RR_p1_)(95% CI) | Difference in AR%  (AF_p2_ – AF_p1_) |
| --- | --- | --- | --- | --- |
| 30 | 1.15 (1.09 – 1.20) | 1.10 (1.05 – 1.16) | 0.96 (0.89– 1.02) | -4.0 |
| 31 | 1.20 (1.13– 1.28) | 1.16 (1.09 – 1.23) | 0.97 (0.89– 1.05) | -2.9 |
| 32 | 1.27 (1.17 – 1.37) | 1.23 (1.14 – 1.32) | 0.97 (0.87– 1.08) | -2.6 |
| 33 | 1.35 (1.22 – 1.49) | 1.32 (1.19 – 1.44) | 0.98 (0.85– 1.12) | -1.7 |
| 34 | 1.44 (1.28 – 1.62) | 1.42(1.26 – 1.59) | 0.99 (0.84– 1.16) | -1.0 |
| 35 | 1.55 (1.34 – 1.79) | 1.55 (1.34 – 1.79) | 1.00 (0.81– 1.23) | 0.0 |
| 36 | 1.68 (1.42 – 2.00) | 1.70 (1.43 – 2.02) | 1.01 (0.79– 1.29) | 0.7 |

The reference temperature is 22.5^o^C

Supp. Table 3. Lag 7 days sensitivity analysis for relative risk, relative risk ratio, differences in attributable risks (AR), and attributable risk percentage (AR%) of temperature-mortality between two periods

| Temperature  (°C) | Period 1  (1992-2009)  RR_p1_ (95% CI) | Period 2  (2010-2019)  RR_p2_ (95% CI) | Relative Risk Ratio  (RR_p2_/RR_p1_)(95% CI) | Difference in AR%  (AF_p2_ – AF_p1_) |
| --- | --- | --- | --- | --- |
| 30 | 1.21 (1.14 – 1.28) | 1.09 (1.03 – 1.16) | 0.90 (0.83–0.98) | -9.1 |
| 31 | 1.29 (1.19– 1.40) | 1.16 (1.07 – 1.25) | 0.89 (0.80–1.01) | -8.7 |
| 32 | 1.40 (1.26 – 1.54) | 1.24 (1.12 – 1.37) | 0.88 (0.77–1.02) | -8.7 |
| 33 | 1.51 (1.33 – 1.71) | 1.35 (1.19 – 1.52) | 0.89 (0.75–1.06) | -7.8 |
| 34 | 1.66 (1.42 – 1.94) | 1.47(1.26 – 1.72) | 0.88 (0.71–1.10) | -7.8 |
| 35 | 1.83 (1.52 – 2.22) | 1.63 (1.35 – 1.97) | 0.89 (0.68–1.16) | -6.7 |
| 36 | 2.05 (1.64 – 2.57) | 1.83 (1.46 – 2.29) | 0.89 (0.65–1.23) | -6.2 |

The reference temperature is 22.5^o^C

Supp. Table 4. Lag 14 days sensitivity analysis for relative risk, relative risk ratio, differences in attributable risks (AR), and attributable risk percentage (AR%) of temperature-mortality between two periods

| Temperature  (°C) | Period 1  (1992-2009)  RR_p1_ (95% CI) | Period 2  (2010-2019)  RR_p2_ (95% CI) | Relative Risk Ratio  (RR_p2_/RR_p1_)(95% CI) | Difference in AR%  (AF_p2_ – AF_p1_) |
| --- | --- | --- | --- | --- |
| 30 | 1.24 (1.13 – 1.36) | 1.03 (0.94 – 1.13) | 0.83 (0.73-0.95) | -16.4 |
| 31 | 1.35 (1.20– 1.53) | 1.08 (0.96 – 1.22) | 0.80 (0.67-0.95) | -18.5 |
| 32 | 1.50 (1.28 – 1.75) | 1.15 (0.99 – 1.32) | 0.77 (0.62-0.95) | -20.3 |
| 33 | 1.68 (1.38 – 2.04) | 1.23 (1.02 – 1.47) | 0.73 (0.56-0.96) | -21.8 |
| 34 | 1.91 (1.50 – 2.43) | 1.32(1.06 – 1.65) | 0.69 (0.49-0.96) | -23.4 |
| 35 | 2.20 (1.64 – 2.95) | 1.44 (1.10 – 1.89) | 0.65 (0.44-0.97) | -24.0 |
| 36 | 2.57 (1.80 – 3.65) | 1.59 (1.15 – 2.20) | 0.62 (0.38-1.00) | -24.4 |

The reference temperature is 22.5^o^C
